# Utilizing the RE-AIM framework for a multispecialty Veterans Affairs Extension for Community Healthcare Outcomes (VA-ECHO) program 2018–2022

**DOI:** 10.3389/frhs.2023.1217172

**Published:** 2023-09-13

**Authors:** Elizabeth A. Mattox, Konstantina E. Yantsides, Maureen Wylie Germani, Elizabeth C. Parsons

**Affiliations:** ^1^ Pulmonary, Critical Care and Sleep Medicine Section, Hospital and Specialty Medicine Service, Veterans Affairs Puget Sound Health Care System, Seattle, WA, United States; ^2^ Health Services Research and Development Center for Innovation, Veterans Affairs Puget Sound Health Care System, Seattle, WA, United States; ^3^ Division of Pulmonary, Critical Care, and Sleep Medicine, University of Washington Medical Center, Seattle, WA, United States

**Keywords:** distance learning, education, telemedicine, vulnerable populations, telehealth

## Abstract

VA-ECHO (Veterans Affairs -Extension for Community Healthcare Outcomes) provides live, synchronous, continuing education accredited, case-based learning. Sessions deliver up-to-date, evidence-based, practice-relevant, Veteran-focused learning to healthcare team members. The primary goal of VA-ECHO is to increase Veterans' access to high quality care by improving knowledge and skills among VA care providers. Utilizing the RE-AIM framework, descriptive statistics for 23 VA-ECHO programs regarding program effectiveness, adoption, implementation, and maintenance during a five-year period (2018–2022) are reported. VA-ECHO offered 1,462 sessions and 157,238 contact hours, engaging 17,642 participants from 837 VA-based sites (20% rural-based sites). Effectiveness includes information on number and diversity of programs, as well as reported impact on participants' practice. Adoption includes descriptive statistics, including comparison of growth and change compared to prior years. Implementation describes change in the program over time, including the number of specialties offered, and types of continuing education offered. Maintenance includes a narrative regarding sustainability. The discussion focuses on implementation and maintenance strategies the program has used to address participant and VA needs within the RE-AIM framework, including adjustments to the program, iterative qualitative improvement, sustainment strategies, and opportunities for future evaluation.

## 1. Introduction

In 2011, the Veterans Health Administration (VHA) provided support for 11 Veterans Affairs-Extension for Community Healthcare Outcomes (VA-ECHO) programs [also known as Specialty Care Access Network (SCAN) ECHO], based on Project ECHO (1, 2). Project ECHO, as implemented by Arora et al., demonstrated that primary care providers were able to treat patients with hepatitis C infection safely and effectively through ECHO-based collaboration with specialists. At the time of implementation, the primary objective of VA-ECHO was to increase access to care among underserved and isolated populations (e.g., rural Veterans) through the de-monopolization of specialty knowledge, and the development of specialty expertise among primary care team members and other non-specialty-based care providers, thus extending specialty care outreach and impact. Prior evaluations of VA-based ECHO projects have reported that participation in VA-ECHO improves knowledge, confidence, and skills (3).

VHA provides services to 9.6 million United States Veteran enrollees at 1,303 sites of care annually (4). Veterans living in rural and highly rural areas are more likely to enroll in VA services than their urban counterparts (58% compared to 38%) (5). Furthermore, rural Veterans enrolled with VA are older and more medically complex than their urban counterparts (5).

Our VA-ECHO program, first implemented in the Northwest Veterans Integrated Service Network (VISN) 20 region, initially focused on five complex chronic conditions: human immunodeficiency virus, suspected lung cancer, chronic hepatitis C, heart failure, and chronic kidney disease (6). Over the years, the regional VISN 20 program evolved to engage a national audience. The initial specialty programs broadened in scope to include general content (e.g., chronic kidney disease expanded to cover general nephrology), and other specialties were added (6). The audience became more diverse, reaching healthcare team members outside of primary care. In 2021, the program transitioned away from regional financial support to national funding sources, and henceforth has referred to itself as Specialty Care VA-ECHO. Other VA-ECHO (and SCAN-ECHO) programs remain operational, including highly regarded local VA-ECHO programs, nationally reaching sub-specialty programs [e.g., inflammatory bowel disease (IBD) VA-ECHO based in Atlanta, GA] and a national Office of Rural Health (ORH) supported Mental Health VA-ECHO based in West Haven, CT. These programs are not included in this analysis.

VA-ECHO offers live, synchronous, continuing education (CE) accredited, case-based learning focusing on evidence-based practice. VA-ECHO facilitates connections between VA specialty care and non-specialty care across large geographic areas, utilizing video-teleconferencing combined with interactive case review. Clinical cases may be integrated within the didactic or presented at the conclusion of the didactic content. Cases are de-identified. Relevant clinical information including diagnostic imaging and laboratory values are presented to the audience. Most programs use the chat function and live polling (multiple choice questions or open-ended questions) to generate engagement from the audience. Some programs have utilized role-play to demonstrate specific skills (e.g., motivational interviewing, trauma informed care). When appropriate, the coordinators distribute additional resources (e.g., national guidelines, low-sodium recipes, patient advocacy organizations, etc.) to learners via email. They may also provide answers to questions that were not answered during the question and answer portion. In some sessions, multidisciplinary panel discussions are utilized. Session duration is 1–1.25 hours. Some programs offer multi-session events, providing 4–6 hours of content per day for 1 to 2 days. Handouts and recordings are available on internal VA SharePoint sites. Sessions are hosted by registered nurse (RN) coordinators and, when possible, utilize regular clinician moderators to introduce the speaker(s), monitor the live chat and relay questions from the audience to the speakers. The specific educational platform utilized has evolved over the years, but during this evaluation period VA-ECHO utilized Microsoft Lync, Skype for Business, and, most recently, Adobe Connect.

Our team previously published an analysis of participation patterns from 2012 through 2018, using similar definitions and methods (6). Supplementary Table S1 integrates the 2012–2018 data. We included data from 2018 in both analyses to permit examination of trends over time, specifically with respect to the COVID-19 pandemic.

## 2. Methods

We employed the RE-AIM framework to describe the program's offerings between January 2018 and December 2022. Our team consulted with the ORH Center for Evaluation of Enterprise-Wide Initiatives (CEEWI) on applying RE-AIM. CEEWI, which represents a collaboration between ORH and VA's Quality Enhancement Research Initiative (QUERI) “supports the ORH's mission by focusing on the implementation, sustainment, and expansion of effective programs to improve the health and well-being of rural Veterans” (7).

The participant cohort in this analysis includes individuals who attended at least one session during the evaluation period. This included healthcare trainees and participants not affiliated with VA. Attendance and completion of brief post session evaluations are optional. The primary incentive for participation is no-cost continuing education (CE) credit. The types of continuing education credit offered for a given session are based on session content, anticipated audience, and available resources. During this evaluation period, 17 forms of CE credit were available (See Supplementary Table S2).

Obtaining CE credit is optional. To obtain CE credit, participants are required to use VA's national online Talent Management System to register for sessions in advance and complete an additional post session evaluation. Some types of CE require additional steps. Our program tracks participants regardless of their intent to earn CE credits or their subsequent completion of these additional steps. With limited exceptions, only VA affiliated participants are eligible to earn CE.

Individuals receiving salary support from VA-ECHO totaling more than 10 h per week (or 25% of full time) were excluded from the analysis. This includes program RN coordinators, and program support, management, and leadership. Medical directors, who receive 0%–20% salary support, are included as participants. They do not receive support specifically for their attendance at sessions, and there are no explicit requirements for them to attend sessions. Faculty are also included in the analysis and do not receive financial support unless they also serve as a medical director.

### 2.1. Determining participant characteristics

We determine participant characteristics primarily through self-report upon initial registration with the program. Participants are asked to provide information including their site, discipline, and clinical specialty when first attending a session offered by our program or when requesting invitations to the sessions. In some cases, we used reputable online sources, including VA-based or academic affiliate faculty directories, or the National Provider Identifier Database (NPID) to address omissions or discrepancies in our data set. For this analysis, we utilized most recently known information for a given participant. Participants were assigned to clinical discipline categories based on the National Uniform Claim Committee (NUCC) provider taxonomy (8) and to a clinical specialty based on the Centers for Medicare and Medicaid Services (CMS) physician specialty codes (9). Healthcare trainees were not assigned to either. Participants were considered rural if they practiced at a location based in a rural, highly rural or insular island community based on the Rural Urban Commuting Area (RUCA) designation as determined by the United States Department of Agriculture (10). When physical location was not clearly identified (e.g., for some participants affiliated with regional and national programs), we considered their location to be urban.

### 2.2. Participant tracking

Several strategies for tracking attendance were utilized during the evaluation period, depending upon educational platforms utilized. Attendance data and evaluation results are maintained in a custom VA Research Electronic Data Capture (REDCap) based project supported by the VA Information Resource Center. We previously utilized a custom MS Access database (3). Quality control measures are regularly applied to ensure data quality.

### 2.3. Evaluations

Responses to brief post session evaluations are collected using internal VA SharePoint and MS Forms and are voluntary. Evaluations are identifiable to permit quality improvement (e.g., analysis by attendee discipline, response to specific concerns). Respondents are aware that their responses are identifiable, however their names are not shared with planning committees or faculty. Evaluations from non-VA participants are not obtained except in very limited circumstances and are excluded in this analysis.

### 2.4. Quality improvement status

VA-ECHO, including iterative evaluation, was approved before implementation as a non-research (quality improvement) initiative. We have secured written verification of non-research status at regular intervals, most recently from the VA Puget Sound Director of Human Research Protection Program and the VA Puget Sound Director of Quality, Safety, and Values. As a designated quality improvement (non-research) project, informed consent is waived and not required.

## 3. Results

### 3.1. Reach

As the goal of our initiative is to improve Veterans' access to high quality care by offering educational development to VA care providers, metrics for the *reach* element of RE-AIM are not included for this analysis, in accordance with guidance from CEEWI. VA-ECHO intends to evaluate *reach* only for sub-projects focused on implementation of specific skills among a limited cohort of care providers whose patient panel characteristics can be described.

### 3.2. Effectiveness

The number and diversity of specialty programs offered and the reported impact on participants' practice are the best measures we have of the effect of VA-ECHO on Veterans' access to high quality care.

#### 3.2.1. Programs

In response to learning needs and emerging clinical issues (e.g., COVID-19), the number of specialty programs offered increased from 12 to 22 during the evaluation period (Table 1). The number of types of CE credits available also increased, from 2 to 16. Specific CE offered varied by program and session based on content, anticipated audience, and available resources.

**Table 1:** VA-ECHO program characteristics (2018–2022).

	2018	2019	2020	2021	2022	2018–2022 combined^a^
Specialty programs	12	12	17	19	22	23
Sessions (% change from prior year)	203 (11%)	210 (3%)	304 (45%)	321 (6%)	424 (32%)	1,462
Types of CE available	2	4	13	16	16	17
Sessions offering CE (%)	202 (99.5%)	209 (99.5%)	275 (90.5%)	321 (100%)	422 (99.5%)	1,429 (97.8%)
Contact hours^b^						
VA-based	6,133	10,202	43,849	43,952	53,102	157,238
Non VA-based	72	44	290	533	326	1,265
Total (% difference from prior year)	6,205 (67%)	10,246 (65%)	44,138 (331%)	44,485 (1%)	53,428 (20%)	158,502
Contact hours/Session	31	49	139	127	108	107
Sessions with case-based learning	–	–	–	62%	57%	–
Unique participants^b^						
VA-based	841	1,581	8,400	7,589	8,791	17,065
Non VA-based	14	15	205	256	141	577
Total (% difference from prior year)	855 (83%)	1,596 (87%)	8,605 (439%)	7,845 (−9%)	8,932 (14%)	17,642
Unique sites^c^						
Rural, highly rural or insular island sites	18	45	94	107	134	168
Urban clinical sites	147	212	392	429	491	580
Other (urban) sites	6	7	30	67	82	89
Total (% difference from previous year)^d^	171	264 (54%)	516 (95%)	603 (17%)	707 (17%)	837
States, territories or districts	48	53	54	53	54	55
Evaluations						
Evaluations returned	1,750	3,226	17,981	24,842	29,768	77,567
Overall evaluation response rate (% difference from previous year)^d^	33%	36.1% (9%)	43.3% (20%)	58.2% (34%)	53.9% (−7%)	47.5%
The content in this session was relevant to my practice (% agree or strongly agree)	94%	92%	90%	90%	90%	90%
I anticipate changing my practice as a result of attending this session (% agree or strongly agree)	77%	75%	74%	76%	77%	76%

^a^ Represents either the cumulative value for entire 5 year period (e.g., unique sites) or total (sum) across the 5 year period (e.g., contact hours).

^b^ Prior to 2020, non-VA participants were rare and not consistently tracked by all programs.

^c^ Includes only VA-based sites, and only one site per participant.

^d^ Some data, including unique sites and evaluation response rates, are not readily available for calendar year 2017, limiting percent difference calculation.

In total, 1,462 sessions were offered during the evaluation period (Table 1). Sessions offered per year varied, from 203 in 2018 to 424 in 2022. The number of sessions and CE offered by a given program are determined by planning committees based on available resources, learning needs, educational content, and anticipated audience. The majority of sessions (97.7%) offered CE; at a minimum, CE for physicians, non-physician providers, and nurses was available.

VA-ECHO offered 39 sessions not specifically affiliated with an existing VA-ECHO program to meet additional learning needs. These included six Veterans Health sessions (sponsored and developed by Pulmonary VA-ECHO); four sessions focusing on virtual care skills, including tele-supervision of trainees during COVID-19 (sponsored and developed by Sleep VA-ECHO); and 29 Chief of Medicine Grand Rounds sessions (provided in support of our local facility during COVID-19). These sessions are included in our analysis (Table 1).

The COVID-19 pandemic resulted in increased demand for virtual learning across the organization. In response, VA-ECHO increased the types of CE offered, the capacity in any given session and the overall scope of offerings while maintaining interactive learning and audience engagement. Furthermore, VA-ECHO focused on more deliberate collaboration with national stakeholders to maintain consistent messaging related to pandemic care.

#### 3.2.2. Session evaluation results

VA-ECHO utilizes brief, standardized post-session evaluations which are optional and identifiable. Evaluation results substantiate that session content is relevant to participant practice (90% or higher) and the majority (76%) anticipate practice change as a result of participation (Table 1). Evaluation response rates are consistently high (average is 47.5%). Evaluations also solicit suggestions for future topics and faculty and are utilized when providing feedback to faculty. De-identified results are reviewed by the multidisciplinary planning committees.

### 3.3. Adoption

#### 3.3.1. Program description

During the evaluation period, VA-ECHO supported 23 different clinical specialty programs (Table 1): Acute Care, Amyotrophic Lateral Sclerosis (ALS), Cancer Rehabilitation, Cardiology, Care Management (care navigation), COVID-19, Diabetes, Endocrinology, Gastroenterology, Healthcare Equity, Hematology, Hepatology, Multiple Sclerosis, Pain Management, Primary Care, Pulmonary, Resuscitation Education and Innovation, Renal, Rheumatology, The Simulation Learning, Evaluation, Assessment, and Research Network Community of Practice, Sleep, Suicide Prevention, and Whole Health.

#### 3.3.2. Participation

**Figure 1:**
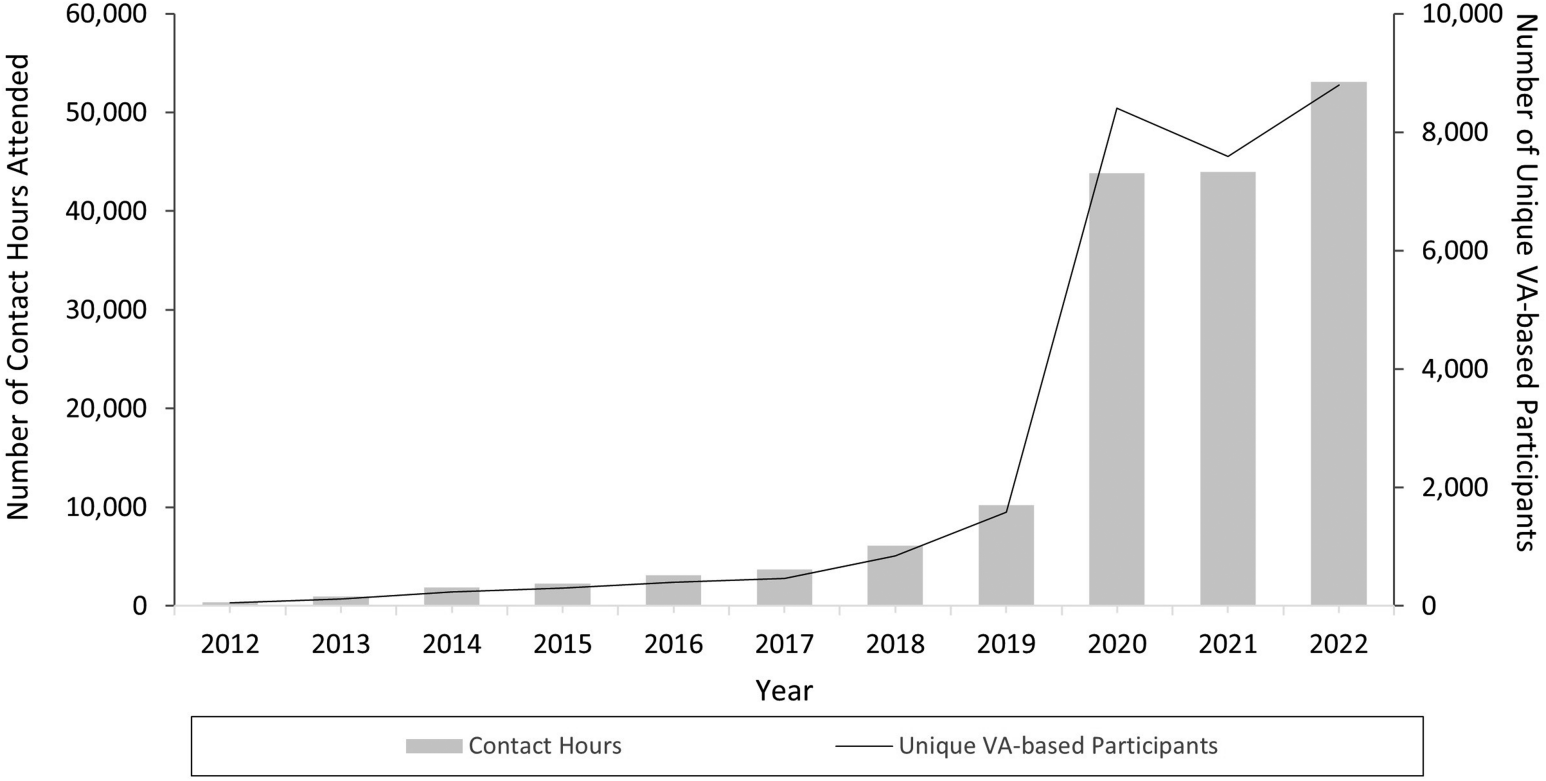
Number of unique VA-based VA-ECHO participants and contact hours provided by calendar year (2012–2022). This figure includes previously published data from 2012 to 2017. There are some small methodological differences between our prior (2012–2018) analysis and this analysis related to exclusion criteria. Specifically unlicensed healthcare trainees, those without clinical licensure including non-clinical staff, and speakers who presented didactic material but did not attend additional sessions as a participant were not included in the prior analysis. The data for contact hours and number of unique participants for 2018 are those values reported in the prior analysis.

During the evaluation period, VA-ECHO provided 158,502 contact hours to 17,642 unique participants, 97% (17,065) of whom were VA-based (Table 1). Contact hours and number of unique participants generally trended upwards year over year (Figure 1 and Table 1), with a noticeable increase between 2019 and 2020. VA-based participants represented 837 unique sites in 55 different states and US territories (Table 1). Of these, 748 (89%) represented clinical sites of care. Of the total sites involved, 20% were located in rural, highly rural or insular island communities. The number of unique sites increased annually from 2018 to 2022. Presuming alignment of the definitions of “clinical site of care” between our analysis and VA published statistics, this demonstrates engagement with over 50% of VA-based clinical sites of care nationally.

#### 3.3.3. Participants

We analyzed VA-based participant characteristics (Table 2). Of the 17,065 VA participants, almost 8% (*n* = 1,284) worked in rural, highly rural, or insular island settings. The vast majority of VA-based participants (93%) were affiliated with a VA-based medical center or clinic.

**Table 2:** VA-ECHO VA-based participant characteristics by rurality (2018–2022).

Variable	Value	RURAL (*n* = 1,284), *n* (%)	URBAN (*n* = 15,780), *n* (%)	TOTAL (*n* = 17,064), *n* (%)
Site type				
	Medical center^a^	982 (76%)	13,082 (83%)	14,064 (82%)
	Community based clinic^b^	300 (23%)	1,628 (10%)	1,928 (11%)
	Regional or national program^c^	0 (0%)	303 (2%)	303 (2%)
	Other^d^	2 (0%)	767 (5%)	769 (5%)
Clinical discipline				
	Behavioral Health & Social Service Providers^e^	277 (22%)	2,843 (18%)	3,120 (18%)
	Pharmacy Service Providers	220 (17%)	2,626 (17%)	2,846 (17%)
	Other^f^	210 (16%)	2,565 (16%)	2,775 (16%)
	Registered Nurses	236 (18%)	2,525 (16%)	2,761 (16%)
	Physicians^g^	106 (8%)	1,852 (12%)	1,958 (11%)
	Healthcare trainees	100 (8%)	1,432 (9%)	1,532 (9%)
	Rehabilitative & Restorative Service Providers^h^	67 (5%)	1,118 (7%)	1,185 (7%)
	Advanced Practice Nurses	68 (5%)	819 (5%)	887 (5%)
Clinical specialty				
	Specialty Medicine^i^	127 (10%)	2,118 (13%)	2,245 (13%)
	Pharmacy	167 (13%)	2,037 (13%)	2,204 (13%)
	Primary Care	205 (16%)	1,820 (12%)	2,025 (12%)
	Other Clinical	128 (10%)	1,751 (11%)	1,879 (11%)
	Mental and Behavioral Health	149 (12%)	1,551 (10%)	1,700 (10%)
	Social Work	145 (11%)	1,413 (9%)	1,558 (9%)
	Healthcare Trainee	98 (8%)	1,390 (9%)	1,488 (9%)
	Nursing	90 (7%)	1,068 (7%)	1,158 (7%)
	Physical Medicine & Rehabilitation	62 (5%)	1,092 (7%)	1,154 (7%)
	Non-clinical	39 (3%)	614 (4%)	653 (4%)
	Administration, Management, Leadership	39 (3%)	573 (4%)	612 (4%)
	Long Term and Extended Care	35 (3%)	353 (2%)	388 (2%)

^a^ Defined as providing at least 2 categories of care (outpatient, inpatient, residential and/or institutional extended care), including Health Care Centers (HCCs).

^b^ Includes community-based outpatient clinics (CBOCs); Other Outpatient Services (OOS) designated sites; multi-specialty CBOCs which may provide ambulatory surgery and/or invasive procedures but which are not HCCs.

^c^ Includes regional Clinical Resource Hubs (CRH), Veterans Benefits Administration (VBA), and other national and regional offices and programs.

^d^ Includes Vet Centers and Community Resource and Referral Centers (CRRCs), stand-alone extended care sites, residential care sites, and those site where site type was not identifiable.

^e^ Includes social workers and psychologists.

^f^ Includes wide range of clinical disciplines including: doctors of chiropractic medicine, dental care providers (DDS, DMD), respiratory therapists, clinical chaplains, etc.

^g^ Includes medical doctors (MD and MB BS prepared), podiatrists and doctors of osteopathy (DO).

^h^ Includes occupational therapists, physical therapists, audiologists, occupational and physical therapy assistants, and speech language pathologists.

^i^ Includes medical specialties (e.g., pulmonary, neurology, cardiology, nephrology and infectious disease).

Participants represent a wide range of clinical disciplines (e.g., nurse, physician, therapist) and clinical specialties (e.g., primary care, pulmonary). Approximately 9% of participants were healthcare trainees and were categorized as such as opposed to a specific clinical discipline or specialty. The most frequently represented disciplines were behavioral health and social service providers (18% of participants), pharmacy service providers (17%), registered nurses (16%), physicians (11%), rehabilitation and restorative care providers (7%), and advanced practice nurses (5%). Behavioral health and social service providers were predominantly social workers (67%, or 13% of all participants) and psychologists (32%, 6% of all participants).

The most frequently represented clinical specialties were specialty medicine (13%), pharmacy (13%), primary care (12%), mental and behavioral health (10%), social work (9%), and physical medicine and rehabilitation (7%). Among specialty medicine, a wide range of specialties were represented including pulmonary (excluding sleep medicine) (41% of specialty medicine, or 5% of all participants), followed by neurology (7%), pain, cardiology, nephrology, and infectious disease (6% each). Of note, pulmonary (including critical care) providers were highly engaged in the initial COVID-19 VA-ECHO program, which was focused on acute and critical care which contributes to their high level of representation. Overall, the distribution of represented disciplines and specialties remains consistent among both rural and urban participants.

#### 3.3.4. Longitudinal attendance patterns

Individual participants attended 1–622 sessions (mean 9; median 2) (Table 3). Over one third [36% (*n* = 6,482)] demonstrated “high” or “very high” participation, attending 5 or more sessions. Approximately one quarter of participants (*n* = 4,352) attended 2–4 sessions (defined as “intermediate participation”) and over one third (*n* = 6,482) attended a single session (defined as “low participation”).

**Table 3:** Specialty care VA-ECHO attendance patterns for VA-based participants (2018–2022).

Results	
Number of attended sessions per participant	
Mean (SD)	9 (22.8)
Median	2
Range	1–622
Number of specialties attended per participant^a^	
Mean (SD)	2.2 (2.3)
Median	1
Range	1–21
Period of attendance (months), *n* (%)	
Less than 1 month	8,137 (48%)
1 to 6 months	2,227 (13%)
6 to 12 months	1,684 (10%)
1 year to 3 years	4,330 (25%)
More than 3 years	686 (4%)
Extent of participation (number of sessions), *n* (%)	
Low (1)	6,482 (38%)
Intermediate (2 to 4)	4,352 (26%)
High (5 to 24)	4,789 (28%)
Very high (25 to 100)	1,294 (8%)
Exceptionally high (more than 100)	147 (1%)
Extent of specialty participation (number of participants), *n* (%)^a,b^	
Exclusive (1 specialty)	10,064 (61%)
Narrow (2–3 specialties)	3,994 (24%)
Intermediate (4–5 specialties)	1,237 (7%)
Broad (more than 5 specialties)	1,254 (8%)

SD, standard deviation.

^a^ Excludes attendance from 39 special topics sessions which were not affiliated with a specific VA-ECHO program.

^b^ Excludes the 516 participants who only attended special topic sessions.

Extent of specialty focus varied, with 61% of participants attending one specialty exclusively (*n* = 10,064) and another 24% (*n* = 3,994) attending 2–3 of the 23 specialties offered. A minority (8%) attended sessions in more than 5 specialties.

### 3.4. Implementation

#### 3.4.1. Changes in program over time

VA-ECHO continually increased the number of specialties, sessions, contact hours and CE types during the evaluation period (Table 1 and Figure 1). Compared with prior analysis covering 2012–2018, the audience is more diverse and, overall, session attendance is markedly higher (6). Prior to the COVID-19 pandemic (March 2020) VA-ECHO was actively expanding, however the pandemic resulted in significantly increased emphasis on virtual learning and created new learning needs. COVID-19 related content (first session: February 2020 in Pulmonary VA-ECHO) and COVID-19 VA-ECHO (first session: May 2020), attracted new participants and a more diverse audience. Applications for more CE types had been submitted prior to pandemic declaration; this expansion was well timed to meet the needs of pandemic-era attendees who were experiencing vastly decreased opportunities for in-person training.

#### 3.4.2. Iterative improvement strategies

Throughout program implementation, VA-ECHO utilized continuous quality improvement strategies to increase program offerings, enhance audience diversity and improve overall program quality and efficiency. Program offerings were increased with respect to number of specialty programs and sessions offered annually (Table 1). VA-ECHO identified learning needs based on program evaluation results, formal learning needs assessments, established and emerging enterprise level initiatives and priorities (e.g., pain management, suicide prevention), and stakeholder input. Stakeholders were identified by planning committee members and coordinators and engaged through direct communication. Once potential new specialties were identified, VA-ECHO offered pilot sessions, and when supported by audience response, implemented new programs as quickly as possible. When necessary, VA-ECHO redistributed workload among RN coordinators to support rapid development and implementation of new programs, often being among the earliest VA-based educational offerings in specific content areas [e.g., Cancer Rehabilitation (11) and COVID-19]. Despite concerns about potential negative impact on existing programs when adding new programs, VA-ECHO has only observed an increase in attendance and audience diversity across all programs (Table 1).

VA-ECHO, as modeled after Arora et al., initially focused on engagement with primary care team members. While VA-ECHO always welcomed participants from all areas of the organization, eventually programs began proactively engaging more diverse audiences. Strategies to improve audience diversity (and size) included broadening planning committee memberships, expanding CE offerings, actively incorporating evaluation feedback from non-primary care attendees, utilizing VA-based national communication tools, employing micromarketing to engage specific groups of learners, adding specialty programs relevant to broader audiences (e.g., ALS VA-ECHO), and engaging with national partners and programs. Facilitating broad engagement predated the COVID-19 pandemic. As a result, when the COVID-19 pandemic occurred, VA-ECHO had an established framework for offering regular, live, multidisciplinary learning opportunities that were scalable and relevant to a very broad audience. In an effort to meet organizational needs, VA-ECHO adapted to permit larger and more diverse audiences as compared to the original Project ECHO model. In recognition of the value of case-based learning, we actively track case presentations (Table 1), and have implemented real-time clinical case consultation based projects when possible and appropriate (e.g., the Sleep VA-ECHO Nightmare—Image Rehearsal Therapy series). Overall, over 50% of sessions offered in 2021 and 2022 offered case-based learning, and we continue to emphasize case-based learning across all programs.

To ensure consistently high-quality offerings, VA-ECHO established standard operating procedures for regular activities including data collection and communication with participants and faculty. VA-ECHO attempts to use the best available educational platform combined with standardization to ensure both consistency across programs and the ability for RN coordinators to support one another without difficulty. RN coordinators and medical directors regularly share challenges and best practices with one another. Each coordinator supports 3–4 programs, further promoting standardization and adoption of best practices across programs.

Specific strategies which support efficiency and optimal resource utilization include a standard program staffing model based on complexity and session frequency. We establish an RN coordinator—medical director dyad (or triad) for each program. RN coordinators manage the day-to-day program needs, including those which require clinical expertise, without placing excessive burden on medical directors. After a formal orientation period, RN coordinators are expected to complete CE applications, lead planning committees, independently identify needed gaps in curriculum and potential faculty, and analyze and present evaluation and needs assessments results. VA-ECHO provides salary support for program medical directors (0.1–0.2 full time equivalent per program), who may be physicians, nurse practitioners, social workers, etc. Medical directors are charged with developing a vision for their program and partnering with the RN coordinator to implement that vision. VA-ECHO enjoys exceptionally high retention of both coordinators as well as medical directors for many reasons, including support of professional development (e.g., abstracts, scholarly publications, academic promotion materials) and allowing each program autonomy with respect to session frequency, areas of focus, type of CE, new projects and innovations (e.g., the Sleep VA-ECHO Nightmare—Image Rehearsal Therapy series).

### 3.5. Maintenance

The sustainability of VA-ECHO relies on maintaining strong relationships with key stakeholders and funders, as well as with program medical directors and faculty. VA-ECHO relies on funding from several VA-based national offices and programs. Funding agreements require annual renewal to maintain continued funding. Maintaining robust data allows VA-ECHO to demonstrate impact and value to current and potential future funders. Providing standardized reports, including evaluation results and detailed information about attendance patterns, demonstrates organizational stewardship to stakeholders.

VA-ECHO's adaptability is another key component to sustainability. Stakeholders, including but not limited to funders, value the ability of the program to quickly offer high quality CE accredited educational offerings on emerging topics (e.g., COVID-19) and enterprise level priorities. This also ensures continued audience engagement. VA-ECHO proactively identifies and quickly offers content relevant to national initiatives [e.g., employee resilience, Pre-Exposure Prophylaxis (PrEP)] and tracks internal metrics. This allows the program to present data on initiatives to key stakeholders and has proven valuable when cultivating partnerships. The strategy demonstrates awareness of leadership priorities while also communicating the relevance of these priorities to the field in real time.

In an effort to maintain program sustainability and establish partnerships with national programs and offices, VA-ECHO has begun to support some national teams with their regular Community of Practice calls (e.g., Healthcare Equity VA-ECHO). While these differ from learning sessions with didactic content and case-based learning, they generally serve the same audience and integrate well with planned curriculum. VA-ECHO provides high quality, low friction offerings by employing standardized processes, facilitating CE accreditation when appropriate and providing attendance data, which have not always been available to these national teams in the past.

Lastly, VA-ECHO recognizes the importance of valuing all members of the healthcare team. We welcome any individual who contributes to care delivery for Veterans, regardless of role or clinical background. During COVID-19, we observed increased proportion of non-clinical attendees and executive leadership, who were either suddenly engaged in direct healthcare delivery at a level not previously experienced, or who had personal questions related to COVID-19 and/or their own wellness and resilience. VA-ECHO provides program planning committees with detailed analysis on their participants, including rurality, specialty, and discipline (as presented in Table 2), and encourages each program to engage new audiences.

## 4. Discussion

Compared with our prior analysis, VA-ECHO engaged a lower proportion of participants affiliated with primary care (40% in 2012–2018 analysis compared to 12% in 2018–2022) (3). During this time, there was notable expansion into content relevant to more diverse audiences (e.g., Multiple Sclerosis VA-ECHO). The pressures on primary care and provider burnout may reduce their overall engagement with programs such as VA-ECHO and their flexibility to attend live sessions. Published discussions of VA-ECHO engagement with primary care providers support the notion that a significant barrier is lack of protected time supporting participation (3, 12–15).

In many respects, the overall patterns of participation are similar to the prior analysis despite expansion with respect to number of specialty programs. Consistent with our previous analysis, approximately one third of participants attended only a single session, and two thirds engaged with a single specialty program over a five year period (6).

The overall increase in diversity with respect to participant specialty and discipline reflects our continued efforts to offer content relevant to all members of the healthcare team. We provide live educational forums where we strive to ensure all healthcare team members are on equal footing, as members of planning committees, faculty, and audience. Evaluation comments reflect interest and increased understanding of the role of the interdisciplinary colleagues, and the specific VA services that they support, offer, and/or promote.

VA-ECHO has diverged from Arora's original Project ECHO model. Both programs utilize a didactic component, however Project ECHO focuses on real time clinical case review, while VA-ECHO as implemented by our team incorporates case-based learning into presentations but is not generally able to facilitate real time clinical case review. The original Project ECHO model targeted an audience of community-based primary care providers from a specific geographical area. VA-ECHO adapted to meet the needs of VA-based audiences to further de-monopolization of (specialty) knowledge. As VA-ECHO continues to evolve, we anticipate engaging increasingly diverse audiences across the nation and expanding into new specialty areas with the ultimate goal of supporting delivery of the highest quality of care to Veterans.

### 4.1. Limitations

There are limitations with our data related to the longitudinal nature of the project and the change over time in program scale and audience diversity. We initially developed processes around sessions with fewer participants (e.g., less than 20) from more narrow range of disciplines and specialties (e.g., primary care providers). The amount of detail we collect for certain types of participants (e.g., healthcare trainees, non-clinical participants) has changed over time. In addition, we have used multiple educational platforms to provide sessions during this evaluation period, each of which has limitations which directly impact attendance data. Furthermore, there is no up-to-date, centralized source of participant demographics available for our team. Changes in participant characteristics (e.g., relocation, career advancement) are certainly underrecognized. In addition, participants are assigned one primary site, discipline, and specialty when in many cases, participants work at multiple sites, have more than one discipline (e.g., PharmD and MD) or specialty (e.g., Sleep Medicine and Psychology), or serve multiple roles in their organization (e.g., leader and clinician).

There are also limitations with the evaluation data. Because evaluations are identifiable, this may influence the likelihood of response and the response itself. Recall bias is another concern since participants have a week from the end of each session to respond to the evaluation.

Other more general limitations with our analysis include potential discrepancy between recorded attendance and actual engagement with session content. In an effort to engage all levels of participants and provide learning opportunities with minimal barriers to entry, we have chosen to not utilize pre- and post-knowledge assessments unless required by accreditation bodies.

### 4.2. Areas for additional evaluation

Ongoing participation with VA-ECHO and Project ECHO programs results in reported improvements in clinical content mastery including higher self-reported knowledge and competencies, self-efficacy, improved patient access to specialty care and useful inpatient care and treatment (3, 13). Future studies are warranted to further explore these relationships including establishing a dose-response threshold over time.

More sophisticated analysis of participation patterns may yield some additional insight about the VA-ECHO audience and better inform curriculum development. Focusing on participants who have been engaged with a single specialty over a period of years and changes to their knowledge, skills, and confidence is another area of potential interest. Ideally, VA-ECHO would directly correlate participation with improved patient outcomes, however that has proven quite difficult for a variety of reasons, including the expansion into new content and audiences.

We are also interested in the effect of participation on participants, medical directors and faculty, including recruitment, retention, and overall job satisfaction. Based on program evaluations, many participants, faculty, and planning committee members report that participating in VA-ECHO and interacting with colleagues throughout the nation results in overall sense of being part of a larger community in support of shared mission and vision for excellence in Veteran healthcare delivery.

Further evaluation about how individual program design influences participation and the benefits is warranted. VA-ECHO supports heterogeneity among programs, however this limits comparison between programs. Alternatively, it may provide an opportunity to examine the impact of program differences including frequency of sessions, utilization of clinician moderators, formats for case-based learning etc.

## 5. Conclusion

VA-ECHO has flourished over the last decade due to the continued support of local, regional and national programs and leadership who support the de-monopolization of knowledge, ongoing professional development and multidisciplinary learning environments. As VA-ECHO continues to evolve, we anticipate engaging increasingly diverse audiences across the nation and expanding into new specialty areas with the ultimate goal of supporting delivery of the highest quality of care to Veterans.

## Data Availability

The raw data supporting the conclusions of this article will be made available by the authors, without undue reservation.
