# Supplementary material for: Utilizing the RE-AIM framework for a multispecialty Veterans Affairs Extension for Community Healthcare Outcomes (VA-ECHO) program 2018–2022
Source: Front Health Serv. 2023 Sep 13;3:1217172. doi: 10.3389/frhs.2023.1217172 (PMC10533985; doi:10.3389/frhs.2023.1217172)
Supplement: Supplementary file 1 [file Table1.pdf]

	2012	2013	2014	2015	2016	2017	2018	2019	2020	2021	2022	Total[1]
Specialty Programs	1	4	5	7	9	11	12	12	17	19	22	24
Types of CE[2]	1	1	1	1	1	2	2	4	15	16	16	17
Sessions	31	94	132	145	157	183	203	210	316	317	418	2,206
Contact Hours	394	941	1881	2271	3111	3707	6,319	10,390	44,063	43,803	52,645	169,525
Contact hours per sessions	13	10	14	16	20	20	31	50	139	138	126	-
Unique Participants	47	117	235	307	396	466	856	1,596	8,609	7,798	8,884	-

1. Represents either the cumulative value for entire 5 year period (e.g., unique sites) or total (sum) across the 5 year period (e.g., contact hours).
2. Prior to 2020, CE was tracked by fiscal year (October – September). As a result, information presented in this table for 2012 – 2019 is estimated.

Table 1S. VA-ECHO Program Characteristics (2012 – 2022)
