# Supplementary material for: Utilizing the RE-AIM framework for a multispecialty Veterans Affairs Extension for Community Healthcare Outcomes (VA-ECHO) program 2018–2022
Source: Front Health Serv. 2023 Sep 13;3:1217172. doi: 10.3389/frhs.2023.1217172 (PMC10533985; doi:10.3389/frhs.2023.1217172)
Supplement: Supplementary file 2 [file Table2.pdf]

Supplemental Table S2

Continuing Education (CE) Offered by VA-ECHO

Continuing Education Type	Acronym
Accreditation Council for Continuing Medical Education	ACCME
Accreditation Council for Continuing Medical Education – Non-Physician	ACCME – NP
Accreditation Council for Pharmacy Education	ACPE
American Academy of Physician Assistants	AAPA
American Board of Internal Medicine - Maintenance of Certification	ABIM-MOC
American Dental Association	ADA
American Nurses Credentialing Center	ANCC
American Occupational Therapy Association	AOTA
American Physical Therapy Association	APTA
American Psychological Association	APA
American Speech-Language-Hearing Association	ASHA
Association of Social Work Boards-New York State Education Department-State Board for Psychology	ASWB-NYSED
Commission on Dietetic Registration	CDR
Continuing Respiratory Care Education	CRCE
Federation of Chiropractic Licensing Boards Providers of Approved Continuing Education	PACE
Joint Accreditation for Interprofessional Continuing Education	JA-IPCE
National Board for Certified Counselors	NBCC
